# The Role of Medical Counseling in the Use of Contraceptive Methods: A Cross-Sectional Public Health Study

**DOI:** 10.3390/ijerph23040507

**Published:** 2026-04-15

**Authors:** Fitim Bexhet Alidema, Lirim Mustafa, Arieta Hasani Alidema, Mirlinda Havolli, Fellenza Abazi

**Affiliations:** 1 Department of Pharmacy, Faculty of Medical Sciences, UBT College, 10000 Pristina, Kosovo; fitim.alidema@ubt-uni.net; 2 Department of Pharmacology, Faculty of Pharmacy, UBT College, 10000 Pristina, Kosovo; 3 Department of Nursing, Faculty of Medical, UBT College, 10000 Pristina, Kosovo; arieta.hasani@ubt-uni.net; 4 Faculty of Health Sciences, University of Maribor, 2000 Maribor, Slovenia; mirlinda.havolli@student.um.si (M.H.); fellenza.abazi@student.um.si (F.A.)

**Keywords:** contraceptive methods, medical counseling, reproductive health, public health, cross-sectional study

## Abstract

**Highlights:**

**Public health relevance—How does this work relate to a public health issue?**

- Contraceptive use remains a critical public health issue influencing unintended pregnancy, maternal health, and reproductive autonomy.
- Limited access to structured medical counseling continues to shape contraceptive choices and safety at the community level.

**Public health significance—Why is this work of significance to public health?**

- Regular specialist medical counseling is strongly associated with higher use of modern and medically recommended contraceptive methods.
- Counseling significantly improves continuity, knowledge, and safety of contraceptive use among adults of reproductive age.

**Public health implications—What are the key implications or messages for practitioners, policy makers and/or researchers in public health?**

- Integrating structured contraceptive counseling into routine reproductive health services may improve population-level reproductive outcomes.
- Public health policies should prioritize counseling as a core intervention alongside contraceptive availability.

**Abstract:**

Background: The use of contraceptive methods is a key component of public health and reproductive health, contributing to family planning, maternal well-being, and social stability. However, contraceptive use is often influenced by the availability and continuity of medical counseling. Limited evidence exists on how regular specialist counseling affects informed contraceptive use in real-world community settings. Methods: A cross-sectional study was conducted between January 2025 and January 2026 using a structured questionnaire. A total of 2400 participants aged 18–55 years were included. The study population was divided into two groups: 1000 women who had been regular patients or receiving consultation for at least one year at the Gynecology and Endocrinology Department of the General Hospital in Ferizaj, and 1400 community participants who had not received regular medical counseling related to reproductive health during the previous year. Data were analyzed using descriptive statistics, chi-square tests, and multivariable logistic regression. Results: The prevalence of current contraceptive use was significantly higher among women receiving regular medical counseling compared with those without regular consultations (72.4% vs. 41.8%; *p* < 0.001). Modern contraceptive methods were more frequently used in the counseled group, including oral hormonal contraceptives (38.5%), intrauterine devices (21.4%), and implants (7.8%), whereas condom use (49.3%) and traditional methods (18.4%) predominated among participants without counseling (*p* < 0.001). Use of contraceptives based on medical recommendation was reported by 81.2% of counseled women compared to 29.6% in the non-counseled group (*p* < 0.001). Long-term contraceptive use (≥12 months) was significantly more common among counseled participants (64.9% vs. 33.5%; *p* < 0.001). After adjustment for age, education, and marital status, regular medical counseling was independently associated with higher odds of modern contraceptive use (OR = 3.62; 95% CI: 3.01–4.35; *p* < 0.001). Conclusions: Regular medical counseling by gynecologists and endocrinologists is strongly associated with informed, consistent, and modern contraceptive use among adults aged 18–55 years. These findings underscore the importance of strengthening structured counseling services as an integral component of public health strategies aimed at improving reproductive health outcomes.

## 1. Introduction

Contraceptive use remains a fundamental component of public health strategies aimed at improving reproductive health outcomes, reducing unintended pregnancies, and promoting maternal and social well-being. Despite the widespread availability of modern contraceptive methods, their effective and informed use varies considerably across populations, influenced by socioeconomic factors, health system performance, and access to professional medical guidance [1,2].

Beyond health system performance and professional guidance, contraceptive behavior is strongly shaped by sociodemographic determinants. Educational attainment, marital status, employment, and broader socioeconomic position have consistently been identified as key predictors of contraceptive uptake, method selection, and continuity of use. Women with higher levels of education are generally more likely to use modern contraceptive methods, partly due to greater health literacy and improved engagement with preventive health services. Similarly, marital status and reproductive intentions influence both the timing and duration of contraceptive use. Recent public health research has further emphasized that educational level, socioeconomic status, and access to structured counseling services represent important predictors of modern contraceptive adoption and continuation rates [3,4].

Inadequate counseling and fragmented reproductive health services with restricted access to implants and intrauterine devices (IUDs) continue to pose challenges to the optimal utilization of contraception, particularly in community-based settings. Recent evidence suggests that the mere availability of contraceptive methods does not guarantee their appropriate or sustained use. Studies have shown that individuals who lack structured medical counseling are more likely to rely on traditional or less effective methods, discontinue use prematurely, or misuse hormonal contraceptives without adequate understanding of indications and contraindications [3,4]. These patterns contribute to persistent gaps in reproductive health outcomes and place additional burdens on health systems.

Sociodemographic and structural determinants also interact with access to services. Individuals with stable employment and higher socioeconomic status may face fewer financial and logistical barriers to obtaining contraceptive counseling and long-acting reversible methods. In contrast, socially vulnerable groups may rely more heavily on informal sources of information or short-term methods due to limited access to structured counseling and specialist services. These considerations underscore the importance of examining counseling effects within a broader social and structural framework.

Medical counseling provided by trained professionals, including gynecologists, endocrinologists, general practitioners, and other reproductive health providers, plays a crucial role in facilitating informed contraceptive choices. Counseling enables individuals to understand the benefits, risks, duration of use, and potential side effects of different contraceptive methods, thereby supporting adherence and long-term use [2,3]. In public health terms, structured and continuous counseling represents a cost-effective intervention with the potential to significantly improve reproductive health indicators at the population level.

However, existing literature reveals notable variability in how counseling services are delivered and accessed. While several studies have examined contraceptive prevalence and method choice, fewer have focused on the comparative impact of regular specialist counseling versus the absence of continuous medical guidance within the same community context [5,6]. This gap is particularly evident in settings where health care utilization is uneven and preventive reproductive services are underutilized.

Given the multidimensional determinants of contraceptive behavior, it is essential to assess whether the observed associations between counseling and contraceptive use persist after accounting for key sociodemographic characteristics. Integrating these determinants into the analytical framework allows for a more nuanced understanding of whether differences between groups reflect counseling exposure, structural access, or underlying social disparities.

Addressing this gap is an important step for informing evidence-based public health policies and optimizing reproductive health services. Understanding whether sustained medical counseling leads to measurable differences in contraceptive use patterns, method selection, and duration of use can guide the design of targeted interventions and strengthen primary and specialist care integration.

Therefore, the aim of the present study was to assess the role of regular medical counseling in the use of contraceptive methods among adults aged 18–55 years. By comparing individuals who received continuous counseling from gynecologists and endocrinologists with those who did not receive regular medical consultations, this study seeks to provide empirical evidence on the public health value of structured reproductive health counseling.

## 2. Materials and Methods

### 2.1. Study Design and Setting

A cross-sectional analytical study was conducted between January 2025 and January 2026. The study was carried out in an urban community setting and within specialist outpatient services, focusing on reproductive health counseling and contraceptive use. Data collection was performed using a structured, self-administered questionnaire administered to eligible participants during the study period.

### 2.2. Study Population and Sampling

The study population consisted of adults aged 18–55 years residing in the study area during the data collection period. A total of 2400 participants were included and stratified into two distinct groups based on their exposure to regular specialist medical counseling related to reproductive health.

Counseled group (*n* = 1000): Women who had been registered patients at the Gynecology and Endocrinology Department of the General Hospital in Ferizaj for at least one year prior to enrollment. Regular counseling was defined as having attended follow-up visits within that one-year period, during which reproductive health and contraceptive use were discussed as part of routine clinical care. The department provides outpatient services for a range of gynecological and endocrine conditions as well as routine reproductive health consultations. Participants in this group were not selected based on any specific medical diagnosis but on documented continuity of care within the department.

Non-counseled group (n = 1400): Community-based participants recruited from the same urban area who reported not having received structured or continuous specialist medical counseling related to reproductive health during the preceding one-year period. These participants were recruited through community outreach in neighborhood settings and public spaces, and eligibility was confirmed through self-report regarding the absence of regular specialist consultations.

Participants were recruited using a stratified approach to ensure adequate representation of both groups. Inclusion criteria were age between 18 and 55 years, residency in the study area, voluntary participation, and sufficient literacy to read and complete the questionnaire independently. Women who were pregnant at the time of the survey were excluded from participation, as their contraceptive needs differ from those of the general reproductive-age population.

Individuals with incomplete questionnaires were excluded from the final analysis.

In the study setting, access to contraceptive methods is available through both hospital-based specialist services and primary care providers. Oral contraceptives are available in community pharmacies, typically requiring medical prescription, while intrauterine devices and implants are primarily inserted in specialist outpatient clinics. General practitioners may provide counseling; however, long-acting reversible contraceptives are most commonly provided by gynecologists within hospital or specialist settings. These contextual factors were considered when interpreting differences between groups.

### 2.3. Data Collection Instrument

Data were collected using a structured, self-administered questionnaire developed based on recent literature and international reproductive health guidelines. The questionnaire comprised four sections:

1. Sociodemographic characteristics (age, education, marital status, employment);
2. Reproductive health history and prior medical consultations;
3. Use of contraceptive methods, including type, duration, and source of recommendation;
4. Knowledge and perceptions related to contraceptive safety and effectiveness.

Participants in the counseled group were additionally asked about the frequency of their specialist visits within the preceding year to confirm ongoing engagement with reproductive health services. The questionnaire also included items regarding the source of contraceptive information (specialist, general practitioner, pharmacist, media, or informal sources) to better characterize exposure to counseling.

Before the main study was conducted, the questionnaire was pilot-tested (beta-tested) on a small group of participants to assess clarity, readability, and comprehension. Feedback obtained during this pilot phase was used to refine the wording and structure of the final questionnaire.

### 2.4. Variables and Outcome Measures

The primary outcome variable was current use of contraceptive methods (yes/no). Secondary outcomes included type of contraceptive method used (modern vs. traditional), use based on medical recommendation, and duration of contraceptive use (≥12 months vs. <12 months).

The main independent variable was regular specialist medical counseling, defined as at least one year of continuous follow-up within the Gynecology and Endocrinology Department, with documented discussion of reproductive health during clinical encounters.

Covariates included age, educational level, marital status, and employment status.

For clarity, modern contraceptive methods were defined as hormonal oral contraceptives, intrauterine devices (IUDs), implants, and male condoms, whereas traditional methods included withdrawal and calendar-based methods.

Irregular contraceptive use referred to inconsistent use of the chosen method (e.g., missed oral contraceptive doses without compensation or inconsistent condom use). The term “unsafe use” was avoided to prevent potentially judgmental interpretation. The term “continuous use” has been replaced with “ongoing use” to better reflect sustained but not uninterrupted utilization.

### 2.5. Statistical Analysis

Data were analyzed using statistical software. Descriptive statistics were used to summarize participant characteristics and contraceptive use patterns. Categorical variables were presented as frequencies and percentages, while continuous variables were summarized using means and standard deviations.

Comparisons between counseled and non-counseled groups were performed using chi-square tests for categorical variables. To identify factors independently associated with modern contraceptive use, multivariable logistic regression analysis was conducted, adjusting for potential confounders. Results were reported as odds ratios (ORs) with 95% confidence intervals (CIs). A *p*-value of <0.05 was considered statistically significant.

### 2.6. Ethical Considerations

Participation in the study was entirely voluntary. All participants were informed about the purpose, procedures, and confidentiality of the study, and written informed consent was obtained prior to questionnaire completion. Participants were assured that their responses would remain anonymous and that they could withdraw from the study at any time without any consequences.

In addition to participant consent, the study protocol was reviewed and approved by the Ethics Committee of the General Hospital in Ferizaj, in accordance with institutional and national ethical standards for research involving human participants. All data were collected and processed in compliance with ethical principles and regulations governing human research.

## 3. Results

A total of 2650 individuals were initially approached and invited to participate in the study. Of these, 120 declined participation, 68 were excluded because they did not meet eligibility criteria (including pregnancy at the time of the survey or insufficient literacy to complete the questionnaire), and 62 questionnaires were excluded due to incomplete responses. The final analytical sample therefore consisted of 2400 participants, corresponding to a response rate of 90.6%.

A total of 2400 participants were included in the final analysis, comprising 1000 women in the counseled group and 1400 in the non-counseled group. The overall response completeness was high, and no substantial data loss was observed following exclusion of incomplete questionnaires. Baseline analyses were first conducted to assess the comparability of the two groups with respect to key sociodemographic characteristics.

Subsequent analyses evaluated differences in the prevalence of current contraceptive use, type of method selected, duration of use, level of knowledge, and patterns of irregular use between groups. These comparisons were followed by multivariable logistic regression modeling to determine whether regular specialist counseling remained independently associated with modern contraceptive use after adjustment for relevant sociodemographic covariates.

The results are presented sequentially to reflect this analytical framework, beginning with descriptive characteristics and progressing to outcome comparisons and adjusted associations.

The results are presented sequentially to reflect this analytical framework, beginning with descriptive characteristics and progressing to outcome comparisons and adjusted associations. The sociodemographic characteristics of the study participants are presented in Table 1.

## 4. Discussion

The present study suggests a strong association between regular medical counseling and contraceptive use patterns among adults aged 18–55 years. By comparing individuals who received ongoing specialist counseling with those who did not, this analysis highlights significant differences in prevalence, method choice, duration of use, and patterns of inconsistent contraceptive use, all of which have important public health implications. Given the cross-sectional design, these findings should be interpreted as associative rather than causal.

### 4.1. Prevalence of Contraceptive Use and the Impact of Counseling (Table 2)

The markedly higher prevalence of contraceptive use among counseled participants (72.4% vs. 41.8%, Table 2) demonstrates a strong association between regular medical counseling and active contraceptive use. Similar associations have been reported in large population-based studies, where counseling was identified as a critical determinant of contraceptive uptake and continuation of use [1,2]. Previous international studies have also demonstrated that structured contraceptive counseling significantly increases both initiation and continuation rates of modern contraceptive methods by is associated with improved continuation of the chosen contraceptive method and the use of more effective contraceptive methods over time [5,7].

**Table 2:** Prevalence of contraceptive use by counseling status.

Contraceptive Use	Counseled (%)	Non-Counseled (%)	*p*-Value
Current use	72.4	41.8	<0.001
Non-use	27.6	58.2	<0.001

However, it is important to consider that access to specialist services may also facilitate access to modern contraceptive methods, particularly long-acting reversible contraceptives that are typically provided in specialist outpatient settings in the study area [4]. Therefore, the observed differences likely reflect the combined influence of structured counseling and facilitated access to contraceptive provision.

### 4.2. Method Selection and Modern Contraceptive Use (Table 3)

Differences in contraceptive method selection between the two groups were pronounced (Table 3). Women receiving specialist counseling were more likely to use modern, highly effective methods such as oral hormonal contraceptives, intrauterine devices, and implants, whereas condom use and traditional methods predominated among non-counseled participants [4,7]. In addition, the proportion of contraceptive use based on medical recommendation was substantially higher among counseled participants compared with those who did not receive regular medical counseling (Table 4).

**Table 3:** Types of contraceptive methods used.

Method	Counseled (%)	Non-Counseled (%)	*p*-Value
Oral hormonal	38.5	26.1	<0.001
IUD	21.4	6.2	<0.001
Implants	7.8	2.1	<0.001
Condoms	27.6	49.3	<0.001

In the local health system context, intrauterine devices and implants are most commonly inserted in specialist clinics, which may partially explain their higher prevalence in the counseled group. Thus, while counseling appears to play a central role in informed method selection, differential access to specialist-delivered services should also be considered when interpreting these findings (Table 5).

### 4.3. Knowledge Levels and Patterns of Inconsistent Use (Table 6 and Table 7)

Knowledge regarding contraceptive methods was markedly higher among counseled participants (Table 6), with a corresponding reduction in inconsistent contraceptive use (Table 7). In this study, inconsistent use refers to irregular application of the chosen contraceptive method, such as missed oral contraceptive doses without appropriate follow-up or inconsistent condom use. The term “unsafe use” was avoided in order to prevent potential misinterpretation that contraceptive methods themselves pose inherent health risks [5,7].

**Table 6:** Knowledge level regarding contraceptive methods.

Knowledge Level	Counseled (%)	Non-Counseled (%)	*p*-Value
Good/Very good	78.6	46.2	<0.001
Poor/Moderate	21.4	53.8	<0.001

The distribution of irregular contraceptive use between counseled and non-counseled participants is presented in Table 7.

**Table 7:** Irregular or unsafe contraceptive use.

Use Pattern	Counseled (%)	Non-Counseled (%)	*p*-Value
Irregular use	12.7	34.9	<0.001
Regular use	87.3	65.1	<0.001

These findings support existing literature indicating that high-quality contraceptive counseling promotes more consistent contraceptive use and continuation over time [5,7].

### 4.4. Independent Predictors of Modern Contraceptive Use (Table 8)

Multivariable logistic regression analysis (Table 8) demonstrated that regular medical counseling remained a strong independent predictor of modern contraceptive use (OR = 3.62; 95% CI: 3.01–4.35), even after adjusting for sociodemographic factors.

**Table 8:** Logistic regression analysis for modern contraceptive use.

Variable	OR	95% CI	*p*-Value
Medical counseling	3.62	3.01–4.35	<0.001
Higher education	1.88	1.45–2.31	<0.001
Married status	1.42	1.10–1.83	0.007

Nevertheless, residual confounding cannot be fully excluded, as individuals engaged in regular specialist follow-up may differ in unmeasured characteristics such as health-seeking behavior, health literacy, or underlying medical conditions.

### 4.5. Public Health Implications

Taken together, these findings suggest that structured and ongoing medical counseling should be considered an integral component of reproductive health services. Importantly, the findings of this study do not diminish the established role of multidisciplinary teams—including general practitioners, nurses, medical assistants, and community health workers—in contraceptive counseling. Rather, they highlight the added value of sustained specialist engagement within the local healthcare context studied. Effective contraceptive care is likely best achieved through coordinated, team-based approaches that integrate counseling with equitable access to contraceptive provision.

In addition, strengthening provider education and training in contraceptive counseling may expand the pool of health professionals capable of delivering structured reproductive health guidance. Policies that support continuing professional education and appropriate reimbursement for counseling services could further encourage healthcare providers to integrate contraceptive counseling into routine clinical practice. Such strategies may improve access to high-quality counseling and ultimately contribute to improved reproductive health outcomes at the population level.

## 5. Conclusions

This study suggests that regular medical counseling is strongly associated with informed, consistent, and modern contraceptive use among adults aged 18–55 years. Individuals who received ongoing counseling from gynecologists and endocrinologists demonstrated higher prevalence of contraceptive use, greater reliance on medically recommended methods, longer duration of use, and safer contraceptive practices compared with those without regular specialist consultations.

The findings indicate that counseling represents an important component of effective reproductive health behavior, beyond the mere availability of contraceptive methods. The association between counseling and modern contraceptive use remained statistically significant after adjustment for key sociodemographic factors, supporting the relevance of structured counseling within reproductive health services.

From a public health perspective, improving access to regular medical counseling within both primary and specialist care settings may contribute to more informed contraceptive choices, improved continuity of use, and enhanced reproductive autonomy. These results support the integration of structured counseling services into broader reproductive health strategies while acknowledging the role of multidisciplinary care models in delivering comprehensive contraceptive services.

Future public health initiatives and policies should emphasize the expansion and standardization of contraceptive counseling, alongside efforts to address educational, social, and structural barriers that may limit equitable access to professional guidance. Such approaches are likely to contribute to the reduction in unintended pregnancies and to the promotion of sustainable reproductive health practices.

### 5.1. Strengths and Limitations

#### Strengths

A major strength of this study is the large sample size (n = 2400), which enhances the statistical power and reliability of the findings. The inclusion of two clearly defined groups—participants receiving regular specialist medical counseling and those without continuous medical consultations—allowed for meaningful comparisons and robust analytical modeling. The use of a structured questionnaire and multivariable logistic regression analysis further strengthened the methodological rigor by enabling adjustment for key sociodemographic confounders.

Another notable strength is the real-world setting of the study, which reflects routine reproductive health practices within both specialist and community contexts. This enhances the public health relevance and external validity of the findings. Additionally, the focus on counseling provided by gynecologists and endocrinologists offers valuable insight into the role of specialist care in shaping contraceptive behaviors.

### 5.2. Limitations

Several limitations should be acknowledged. First, the cross-sectional design precludes causal inference, and associations observed between medical counseling and contraceptive use should be interpreted accordingly. Second, data were collected through self-reported questionnaires, which may be subject to recall bias or social desirability bias. Third, the study was conducted within a single urban setting, which may limit the generalizability of the findings to rural or socioeconomically different populations.

Despite these limitations, the consistency of the results with existing evidence and the magnitude of observed associations suggest that the findings provide meaningful contributions to the understanding of counseling-related determinants of contraceptive use.

## Figures and Tables

**Table 1:** Sociodemographic characteristics of participants (n = 2400).

Variable	Counseled (n = 1000)	Non-Counseled (n = 1400)	*p*-Value
Mean age (years)	33.1 ± 8.4	32.6 ± 8.7	0.21
Higher education (%)	48.2	35.6	<0.001
Married (%)	62.5	54.3	<0.001

**Table 4:** Use of contraceptives with medical recommendation.

Use Based on Recommendation	Counseled (%)	Non-Counseled (%)	*p*-Value
Yes	81.2	29.6	<0.001
No	18.8	70.4	<0.001

**Table 5:** Duration of contraceptive use.

Duration	Counseled (%)	Non-Counseled (%)	*p*-Value
≥12 months	64.9	33.5	<0.001
<12 months	35.1	66.5	<0.001

## Data Availability

The data presented in this study are available from the corresponding author upon reasonable request, due to ethical and privacy considerations.
